# Protocol for assessing the hypotensive effect of evening administration of acetylsalicylic acid: study protocol for a randomized, cross-over controlled trial

**DOI:** 10.1186/1745-6215-14-236

**Published:** 2013-07-27

**Authors:** Mª Victoria Ruíz-Arzalluz, Mª Cruz Gómez Fernández, Natalia Burgos-Alonso, Ernest Vinyoles, Ricardo San Vicente Blanco, Gonzalo Grandes

**Affiliations:** 1 Primary Care Research Unit – Bizkaia, Basque Health Service (Osakidetza), Bilbao, Spain; 2 Primary Care Universitary Research Institut, Jordi Gol Foundation, (IDIAP Jordi Gol), Barcelona, Spain; 3 Primary Care Center of Andoain, Basque Health Service (Osakidetza), Gipuzkoa, Spain; 4 Primary Care Center of Zumarraga, Basque Health Service (Osakidetza), Gipuzkoa, Spain

**Keywords:** High blood pressure, Low-dose ASA, Bedtime administration, Secondary prevention

## Abstract

**Background:**

The objective of this study is to evaluate the antihypertensive effect of bedtime administration of low doses of aspirin in patients with treated hypertension and high cardiovascular risk on low-dose aspirin for secondary prevention, in order to optimize their usual treatment and reduce their cardiovascular risk.

**Methods/Design:**

This is a prospective phase IV multicentre, randomised, triple-blind, placebo-controlled, cross-over clinical trial. We will include 258 individuals with hypertension treated with low-dose aspirin for secondary prevention. These patients will be randomly recruited, by approximately 40 primary care physicians collaborating in the study, mainly in the Guipúzcoa West, Bilbao and Barcelona areas. The 258 patients will be randomly allocated to treatments to create two comparable groups. In the first period, the intervention group will take aspirin at night and placebo in the morning, while the control group will take their aspirin in the morning and placebo in the evening for 2 months. After a washout period of 15 to 30 days, there will be a second 2-month period for which groups will swap treatments. Participants will undergo ambulatory blood pressure monitoring at baseline, at the end of first period and then again at the beginning and end of the second period. The main outcome measure is the change in mean blood pressure over 24 h, and as secondary outcomes we will also assess changes in systolic and diastolic blood pressure, during the day and night, and the relationship between them. Lastly, we will explore whether non-dipper patients convert into dippers with the intervention.

**Discussion:**

The goal of this research is to provide the scientific basis for indicating a change in the time of aspirin administration from morning to evening, by primary health practitioners, to improve the patient control of blood pressure and more effectively reduce their cardiovascular risk, by combining this hypotensive effect with the well-known anti-platelet effect of low-dose aspirin.

**Trial registration:**

ClinicalTrials.gov NCT01741922

## Background

In the last decade, several interesting studies have been published demonstrating the hypotensive effect (7/4 mmHg) of low-dose acetylsalicylic acid (ASA) administered in the evening. These effects are not observed if the ASA is administered at other times of day [1-3]. Further, it has been reported that 58% of non-dipper patients become dippers under evening administration of ASA [2]. These authors attribute the phenomenon to the inhibitory effect of ASA on angiotensin II, by the release of nitric oxide (NO) from the vascular endothelium, being 30% stronger when ASA is administered in the evening [2,3].

To the best of our knowledge, trials reported in the literature have all been carried out in healthy or mildly hypertensive individuals who were not under treatment for hypertension prior to the study [1,3,4]. On the other hand, millions of hypertensive patients across the world take ASA for secondary prevention, usually in combination with other drugs. Despite this, researchers have not included these patients in trials to assess the hypothetical hypotensive effect of bedtime ASA. It therefore remains to be determined whether the observed effect is reproduced in patients with a medical history of cardiovascular events, the very individuals who are often prescribed low-dose ASA [5-8].

The great importance of controlling blood pressure (BP), as a cardiovascular risk (CVR) factor is very widely recognised [9,10]. In addition, it has been demonstrated that morbidity and mortality are lower among individuals whose BP falls more at night [11-13], one study finding that a decrease of 5% in the magnitude of the night-time dip increased mortality by 31% [14]. Despite the broad range of antihypertensive agents available, efforts of healthcare professionals and extensive research on the subject, the control of BP still represents a challenge and is not always achieved. In the 2010 PRESCAP study, only 46% of patients had good control of their systolic and diastolic BP, despite the fact that 63% were on more than one antihypertensive drug [15]. It is, therefore, clear that any drug treatment that could contribute to the control of BP should be investigated and, particularly, when the drug involved, ASA, is already widely used.

If the hypotensive effect of taking low-dose ASA at bedtime were to be confirmed, this would be an important advance for these patients as, without adding new drugs to their regimen (with potential adverse effects) and with minimal inconvenience, we would be able to improve the control of their BP and more effectively reduce their cardiovascular risk by combining this hypotensive effect with the well-known antiplatelet effect of low-dose ASA.

## Methods/Design

### Objectives and hypothesis

#### Objectives

The general objective of the study is to assess the hypotensive effect of administering ASA at bedtime instead of during the day, in hypertensive patients under preventative treatment with low-dose ASA and a history of cardiovascular events. This general objective can be broken down into several specific objectives:

•To quantify the change in blood pressure associated with the bedtime administration of low doses of ASA in hypertensive patients with history of cardiovascular events and who have been regularly taking this drug during the day for secondary prevention

•To measure the night-time dip in BP after bedtime administration of ASA at low doses

•To explore any changes in the circadian pattern of BP, after changing the time when ASA is taken, and the potential transformation of non-dippers to dippers

•To investigate potential differences in the hypotensive effect of bedtime administration of low-dose ASA in various different subgroups by age, sex, serum creatinine levels, et cetera

•To investigate potential differences in the hypotensive effect of bedtime administration of low-dose ASA as a function of the antihypertensive treatment given to patients

•To investigate potential changes in adverse effects when ASA is taken at bedtime rather than during the day

#### Hypotheses

In hypertensive patients on low-dose ASA for secondary prevention, mean BP will fall by at least 5 mmHg if the drug is taken at bedtime rather than at another time of day. Further, these individuals will improve control of their BP, which is one of their main CVR factors.

The percentage fall in mean night-time BP will be higher among non-dippers than among individuals already classed as dippers. The percentage of individuals who are dippers will increase among the patients undergoing the intervention (with the change in the time of day low-dose aspirin is taken).

**Figure 1:**
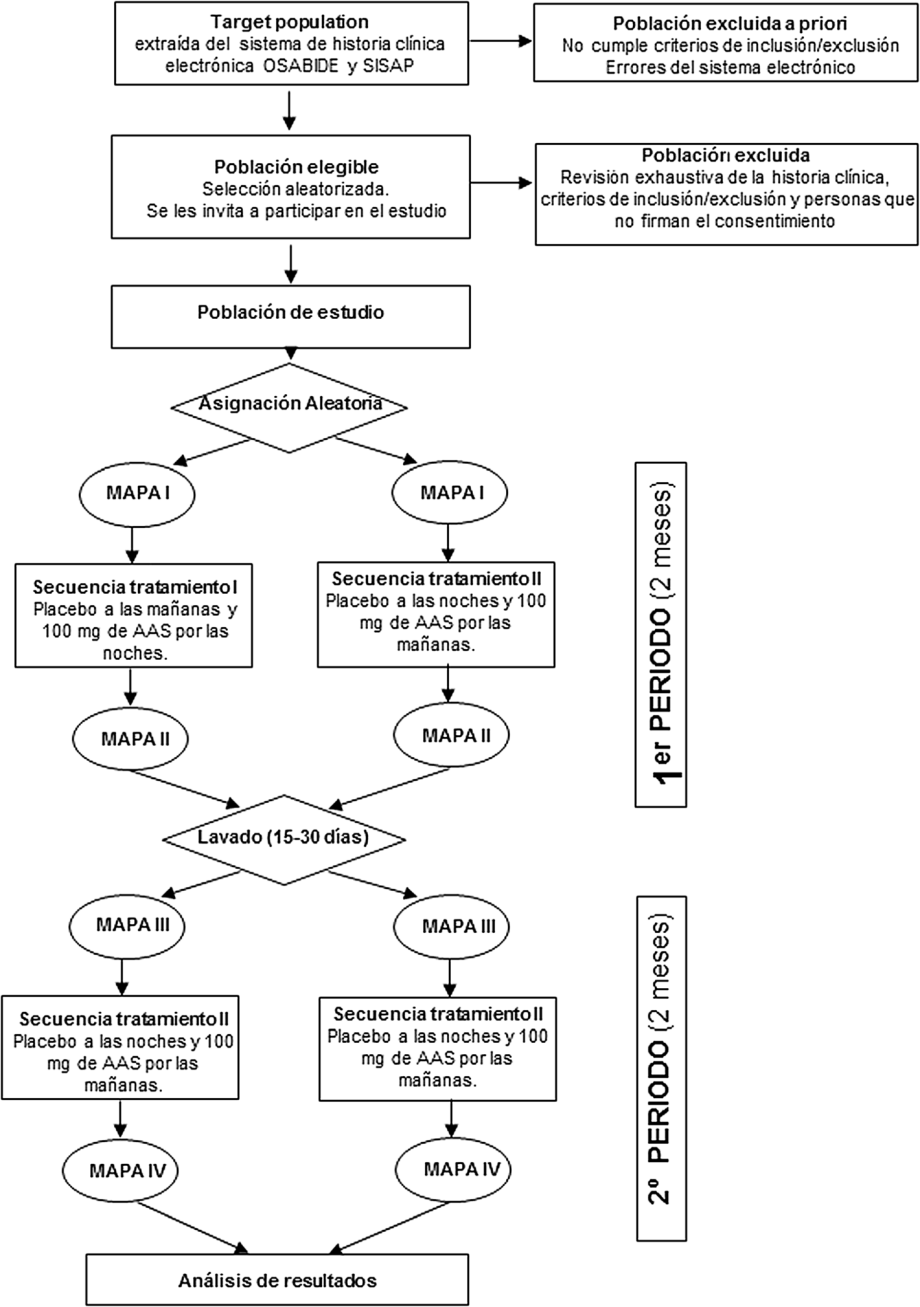
Flow of the study.

At least 40% of non-dippers in the study will become dippers on changing the time of they take their aspirin (Figure 1).

### Study design

This is a multicentre, randomised, triple-blind placebo-controlled, cross-over study in patients with hypertension and a history of cardiovascular events under treatment with low-dose ASA taken during the day time.

Patients will be randomly selected from the target population of each primary health practitioner that collaborates in the study. This first randomization will be performed by the Primary Care Research Unit of Bizkaia (PCRUB). After that, they will be randomly and blindly assigned to two successive sequences of bedtime and daytime administration of ASA, the drug and placebo both being manufactured by Laboratorium Sanitatis. One group of patients will take ASA over a period of 2 months at bedtime and placebo in the mornings, while in the second period they will take ASA in the mornings and placebo at bedtime (BD sequence). The other group will follow the opposite sequence (DB sequence).

On inclusion in the study, all participants will be prescribed the same standard dose of 100 mg ASA/day. Between the two periods there will be a washout period of 15 to 30 days (selecting the most convenient time for each patient, as explained below), during which all patients will take ASA in the mornings. Despite the fact that the half-life of ASA ranges from 2 to 20 h, its potential hypotensive effect may be mediated by mechanisms associated with the physiology of vascular epithelium that may last for much longer (as much as a week), regardless of whether the ASA itself has been cleared from the body. In the light of current knowledge, a washout period of at least 15 days should be sufficient for the effect of the first sequence of treatment not to interfere with that of the second period. The results will be assessed by monitoring BP at baseline and 2, 3 and 5 months after the intervention.

### Setting

The study is intended to be conducted in a total of 20 primary care centres: six in Bizkaia and six in Gipuzkoa, all managed by the Basque Health Service-Osakidetza, and a further eight in Barcelona, managed by Catalan Health Service. In addition, each of the health centres are linked to the PCRUB or the Jordi Gol Foundation (in the Basque Country and Catalonia respectively), as well being members of the Primary Care Research Network (redIAPP) for research into preventative activities health promotion, and of the Consortium to Support Biomedical Research Networks (CAIBER).

The primary health centres in the Barcelona region are: Santa Coloma de Gramanet, el Maresme, Badia del Valles, Gotic, Passeig de Sant Joan, Sant Martí de Provençals, Passeig Maragall and la Mina; the centres in Bizkaia are: el Karmelo, Gasteleku, Areilza, Solokoetxe, Basurto and Kueto; and, finally, those in Gipuzkoa are: Andoain, Oñati, Zumárraga, Tolosa, Ermua and Azpeitia. The doctors and nurses collaborating in the study are all primary care health professionals, and the patients who will participate are those they regularly see in appointments at the health centres.

The health centres have offices for medical and nursing appointments, blood collection facilities and rooms for performing other types of tests, as well as all the other equipment necessary for undertaking this study. Nevertheless, in order not to disrupt the normal operation of the centres in relation to ABPM, all will be provided with the equipment required for performing the BP monitoring necessary for this study.

The PCRUB will be in charge of the coordination of the performance and monitoring of the fieldwork, as well as the quality assurance and control and the statistical analysis of data collected for the study. For this purpose, it has:

•Meeting rooms with informatic support to hold coordination meetings and training sessions for collaborating researchers

•A centralised database, with various levels of access for users from each of the participating health centres, specifically designed for this study

•Licences for software for statistical analysis (SAS)

•Support staff to perform technical and administrative tasks, coordination, monitoring, and quality assurance and control, as well as the statistical analysis of data

### Participants

#### Inclusion criteria

To be eligible for inclusion patients will need to:

•Be aged >18 years of either sex

•Have diagnosis of hypertension

•Have treated hypertension; this is when at least the patient receives one antihypertensive drug

•Be primary care users in which anti-hypertensive drug regimen has not been changed in the last 3 months

•Have a medical history of a cardiovascular event at least 6 months previously (ischaemic heart disease, cerebrovascular accident (CVA), peripheral arterial disease) and have been treated at least for 3 months with low doses of ASA (75–125 mg)

•Sign an informed consent form

•Complete, prior to inclusion, a washout period of 1 month during which they take ASA in the morning, if they had been under regular treatment with ASA at bedtime

#### Exclusion criteria

Patients will be excluded if they meet any of the following criteria:

•Being pregnant or breastfeeding

•Having poorly-controlled high blood pressure, defined as having a systolic BP >150 mmHg or a diastolic BP >95 mmHg in two successive health checks

•Requiring non-steroidal anti-inflammatory drugs (NSAIDs) on a daily basis

•Having a severe or terminal disease

•Being diagnosed with a physical or mental disorder that would hinder their collaboration

•Taking other antiplatelet or anticoagulant agents concomitantly

•Being on ASA at doses outside those stated in the inclusion criteria

•Being a shift worker or having a very intensive night-time work schedule

•Remaining unstable despite the treatment or requiring regular adjustments of the treatment due to their clinical status

•Being a heavy drinker, consuming >280 g of alcohol per week in the case of men or >170 g for women

•Having congestive heart failure or moderate to severe chronic renal failure that is New York Heart Association (NYHA) stage ≥III or a glomerular filtration rate < 45 mL/min

•Patients having low blood pressure ≤ 110/60 mmHg

*Withdrawal criteria*:

•Undergoing changes in their treatment within the 5-month period of the trial

•Requiring admission to hospital during the clinical trial

### Recruitment process

Patients meeting the inclusion criteria will be identified using the electronic medical record systems, OSABIDE in Osakidetza and the SISAP in the Catalan Health Service. Considering the information in the medical record, each doctor will check the list of candidate patients, rejecting those that *a priori* meet any of the exclusion criteria, that is, cases in which inappropriate patients have been identified by the electronic selection process. After that, the trial monitor will review this first filtering by the doctors, to ensure that it has carried out been correctly, and patients will be randomly selected by a computer program in the PCRUB, until seven or eight patients have been included per doctor. A randomly-ordered back-up list of potential replacements will be prepared, to use in the event that any of these seven or eight initially selected patients decline to give informed consent or are excluded for not meeting the selection criteria. If the target population on any doctor’s list is too small, we will ask another doctor from the same health centre, who until that point has not been involved in the recruitment process, to grant us permission to identify patients within the target population on their list, following the aforementioned procedure for potential replacements. Once a patient is included in the study they will receive a letter, with a follow-up telephone call, inviting them to make an appointment with their general practitioner (GP). During this appointment, their doctor will explain the characteristics of the study and invite them to take part. If patients agree and sign the informed consent form, they will be included in the study population. At this point, their doctor will enter them in the electronic data capture (EDC) system, a code being automatically generated for that patient for the study. This code is formed by five digits: .

**A**: The first digit will correspond to the province where the patient is registered.

**A and B**: The first and second digits combined will indicate the patient’s health centre.

**A**, **B and C**: The code identifying doctors and nurses is based on the first three digits of each patient’s code, preceded by a D or N for doctors and nurses, respectively.

**D and E**: The last two digits of the code individually identify each of the patients.

Once the code has been generated, the doctor will enter data regarding patient registration, sociodemographic characteristics and clinical history, and make an appointment for the patient with a nurse.

### Randomisation

Randomisation will be performed in a 1:1 ratio using computer-generated random numbers provided by a central site, the Laboratorium Sanitatis. Specifically, when the medication pack number for each of the 258 patients participating in the study is associated with a single patient, this individual is randomly assigned to one of the two treatments: that is, having placebo at bedtime (administered between 20:00 and 22:00) and 100 mg of ASA in the mornings (administered between 08:00 and 10:00), on the one hand; or placebo in the mornings (administered between 08:00 and 10:00), and 100 mg of ASA at bedtime (administered between 20:00 and 22:00). After the washout period of 15 to 30 days, the patients start their medication for the second 2-month period of the study, and their treatment will have been swapped; that is, those who in the first period had the placebo at bedtime (between 20:00 and 22:00) and 100 mg of ASA in the mornings (administered between 08:00 and 10:00) will have the placebo in the mornings (administered between 08:00 and 10:00) and 100 mg of ASA at bedtime (between 20:00 and 22:00) in this second period, and vice versa.

Throughout the study, the patient’s doctor and nurse and the person carrying out the statistical analysis will be blind to when the patient is taking the active ingredient under study.

### Intervention

#### First period

Patients included in the study will be referred for a nursing appointment by their GP. In this appointment, the nurse will take several anthropometric measurements and measure blood pressure, after patients have rested for 5 min (initially, two readings, but if the difference is >5 mmHg another reading will be taken, and the final measurement will be the mean of the two or three readings obtained). The same day, urine and blood samples will be collected. Later, after explaining to patients what ABPM involves, they will be fitted with a portable blood pressure monitor. Patients will be asked to carry out their normal daily activities with few restrictions and provided with a notebook for them to write down the main events in their day, as well as the time they woke up, went to bed, et cetera.

Twenty-four hours after placing the ABPM device, patients will be seen again by a nurse in their health centre, for the monitoring system to be removed. At this point, the nurse will provide patients with their corresponding medication pack, which will be assigned with the randomisation code and recorded in the EDC system. This pack will consist of a box with two containers, one holding the medication to be taken in the morning and the other that for bedtime. Both containers will have enough pills to complete the entire 2 months of treatment. On completion of the first month, the patients will be seen again by a nurse, for the first follow-up visit. The participants will be reminded of the importance of taking the medication as instructed, that they must bring back the medication containers even if they are empty, and that they should record in their notebook any symptoms that they have at any point during the month that could be adverse effects and the day on which they had them. Finally, a new appointment will be made for the following month, by the nurse.

In the second follow-up visit, the nurse will check patient adherence to the treatment again, by counting the empty blisters and directly asking the patients about their daily intake of the drugs, whether they took the medication at the right time and whether there had been any changes with respect to their initial medication regimen. In the event of non-adherence and/or when new drugs had been introduced, the nurse will contact the research team, to decide whether the patient should continue or be withdrawn from the study. In addition, the nurse will be in charge of investigating whether there had been adverse effects and collect the empty blister packs. In the second follow-up visit, just before completion of the first period, the blood pressure monitor will be fitted on the patient for the second 24 h of ABPM. The following day, the nurse will remove the device, make an appointment for between 15 days and 1 month later, and provide the medication for this time, which corresponds to the washout period (see below). Then the first period of the trial will have been completed (Figure 1).

#### Washout period

Given the cross-over nature of this study, the first and second study periods are separated by a washout period of between 15 and 30 days. This must be of sufficient time for the effect of the drug taken during the first period to disappear and not interfere with the results of the effects of the drug taken in the second period. In this case, the same active ingredient is taken, but at different times of day. During the washout period, all the study patients will be asked to take ASA during the day as they did before the start of the trial. The duration of 15 to 30 days could seem to be excessively long as the half-life of ASA ranges from 2 to 20 h [16] and, indeed, even 1 week should be sufficient time for the drug to be cleared from the body and for any effects of bedtime administration to wear off. Nevertheless, for organisational reasons and so that ABPM measurements are further apart and interfere less with the patients’ daily life, we have opted for a variable washout period.

#### Second period

At the start of the second period, the nurse will set up the ABPM system for the patients for the third time. After removing the ABPM device, 24 h later, patients will be given the medication and instructions on how to take it over the following 2 months and an appointment with a nurse will be made for them the next month, as in the first period. In this second period, patients swap their treatment sequence: that is, those patients who had placebo at bedtime in the previous period (between 20:00 and 22:00) and 100 mg of ASA in the mornings (administered between 08:00 and 10:00) will take placebo in the morning (administered between 08:00 and 10:00) and 100 mg of ASA at bedtime (between 20:00 and 22:00) in this second period and, vice versa, those who in the first period had placebo in the morning (administered between 08:00 and 10:00) and 100 mg of ASA at bedtime (between 20:00 and 22:00) will now take placebo at bedtime (between 20:00 and 22:00) and 100 mg of ASA in the mornings (administered between 08:00 and 10:00). Some days before the end the second month of the second period, a fourth 24-h ABPM measurement will be taken, as will blood and urine samples. When the data from this second period have been transferred to the database and it has been confirmed that the blood test results are within normal ranges or that if there have been changes they are judged not to be clinically relevant by the research team, the trial will come to an end (Table 1).

**Table 1:** Design of study

**Schedule of events (Day counted from the first visit, Day 0)**	***Screening: visit *1 (Day −15)**	***Baseline*, *enrolment, randomization: visit 2 *(Day 0)**	**Treatment: *visit 3 *day 30**	**Treatment *visit 4 *day 60**	**Treatment *visit 5 *day 90**	**Washout 1 month**	**Treatment *visit 6 *day 150**	**Treatment *visit 7 *day 180**	**Treatment *visit 8 *day 210**
**Study procedures**									
Informed consent form	X								
Medical history	X								
Physical examination including weight and height measurements		X							
Demographic data	X								
**Intervention**									
Control/Intervention group I (ASA in the morning and placebo in the evening in the first period)			X	X	X	Crossover	X	X	X
Intervention/Control group II (placebo in the morning and ASA in the evening in the first period)			X	X	X		X	X	X
**Blood chemistry**									
Haematology and biochemistry		X							X
**Efficacy measures**									
ABPM		X			X				X
**Safety assessment**			X	X	X		X	X	X

Each of the patients will be followed up over a period of 5 months.

### Evaluation of results

The primary outcome measure will be the mean blood pressure (MBP), and the secondary outcome measures will be the systolic and diastolic BP, HR and pulse pressure (PP). All of these will be measured over 24-h periods with a clinically-validated ABPM device, Watch BP 03. The research nurses will fit this ABPM device on patients and remove it after the 24-h measurement period, having first measured the patient’s BP with a validated OMRON M6 blood pressure monitor twice, or three times if the difference between the first two measurements is >5 mmHg. After that, they will give patients a notebook to record their activity, exercise and meals times as well as the time they go to bed and wake up during the 24 h of monitoring.

The systolic and diastolic BP as well as the HR will be measured every 20 min between 07:00 and 23:00 and every 30 min between 23:00 and 07:00. Monitoring will not be considered valid if >30% of the data are missing or if there is no data for a period >2 h. It will also not be considered valid if the patient has an irregular sleep-wake rhythm, with resting times >12 h or <6 h per night, during the monitoring period [17]. We will define the daytime BP as the mean of the measurements collected between 09:00 and 21:00 and the night-time BP as the mean of the measurements taken between 0:00 and 06:00, as this is the timetable that most closely matches the habits of most of our patients.

In addition, we will calculate the night/day ratio of the BP and classify patients according to this value ratio : >1, riser; >0.9 and ≤ l, non-dipper; >0.8 and ≤ 0.9 dipper; and ≤ 0.8, extreme dipper.

### Adverse effects

At each appointment with the patients, the nurse, as well as providing the medication for the following period and assessing treatment adherence, will ask the patient to record any AEs detected during the study period. These AEs will also be recorded by the nurse in the dedicated database, establishing their severity and assessing the causality with respect to the drug under study. Further, there will be an independent committee who will monitor the safety of the drug under study, by reviewing any AEs that occur during the entire study period.

In the event of a serious adverse reaction (SAR) or a suspected unexpected serious adverse reaction (SUSAR), it will not be necessary to break the blind nature of the study since the medical actions taken will be the same for all patients, independent of the knowledge of group allocation, since all the patients are taking the same active ingredient and the placebo. The only difference is time of day they are taking the drug. All the current regulations will, in any case, be complied with at all times and in the event of any of the aforementioned types of reaction, the relevant health authorities will be notified within 24 h of them being reported.

### Data quality and management

The coordination, control of quality of processes related to the study, data management and quality assurance will be the responsibility of the PCRUB. The patients will be randomly selected and the reasons for any being excluded will be recorded in their medical records and revised by the trial monitor. Changes in the BP of participants will be assessed by 24-h ABPM, coordinated by nurses trained in the technique, who will be blind to the assignment of patients to the comparison groups, under the supervision of the research team. Moreover, various measures will be taken to guarantee the quality and validity of the data collected in the study including:

•A EDC system will be created for recording data and steps taken to minimise errors in data recording and later processing of the data, such as double-data entry, follow-up of missing data, and automatic transfer of the ABPM data from the device to the EDC system, among others

•Documentation will be completed to ensure the traceability of the drug under study over the entire study period

•Inspections will be made to check that the study is being carried out in accordance with good clinical practices and the established protocol

### Sample size

A sample size of 258 patients, with 129 in each group, will provide a statistical power >80% for detecting as significant (*P*< 0.05) a difference of 2.5 mmHg between the mean arterial pressure with bedtime and daytime administration, using a two-tailed Student’s t-test (standard deviation obtained from previous studies, with similar populations to that of our project and estimated to be 8 mmHg) [4].

A reduction of 5 mmHg in arterial pressure is considered to be clinically significant at the individual level, since it is associated with a decrease of 12.5% in the risk of cardiovascular events and of 16.7% in the risk of acute cerebrovascular accidents [7]. We expect that with bedtime administration at least 50% of patients will achieve this level of reduction in BP, and that there will be no changes during day-time administration. Under this assumption, the mean difference between the two groups would be at least 2.5 mm/Hg.

These calculations are based on the formula for calculating sample sizes in cross-over clinical trials developed by Donner (1984) [18]. It has been assumed that around 5% of the BP measurements will not be valid, that around 15% will be lost to follow-up and that the intra-subject correlation will be 0.5.

### Analysis

First, an assessment will be made of the potential carry over effect of the treatment from the first period to baseline levels before the second period. The analysis will be carried out on an intention-to-treat basis, comparing the mean blood pressure at the end of the period of bedtime intake with that observed after the daytime intake, adjusting for the baseline blood pressure at the start of each period in all patients which at least have received one dose of treatment. Moreover, we will perform a per protocol analysis with only the patients have not change in their treatment within the 5-month period of the trial and have not been withdrawn. The effect attributable to bedtime intake will be estimated in terms of the difference in mean blood pressure between the two periods and the 95% confidence interval will be calculated using mixed-effect models analysis of covariance for repeated measures of blood pressure taken in each subject at the end of each of the periods. These models will include the patient as a random effect (intercept), to take into account the correlation between the two measurements in the same individual in each period. As well as adjusting for the baseline levels, we will adjust for potential confounding or modifying variables: changes in medication, period or sequence, age, sex, unhealthy habits as to be smoker or no smoker, risk factors as to be dipper or non-dipper comorbidity as diagnosis of dyslipemia, diabetes, stroke, peripheric arteriophaty or myocardial infarction and month of the year when the study is started. We will assess, by means of tests of interaction, the absence of a modifying effect of bedtime intake as a function of the period when patients took the ASA at this time.

A stratified analysis will be undertaken considering two groups, dippers and non-dippers, assuming that the prevalence of non-dippers in this population is 40%.

In the mixed-effect analysis, all the data available from each of the subjects included in the study will be considered, regardless of whether there is missing data. Values of missing data will not be imputed, except in the case of the baseline values for the second period, in which case they will be assigned the same value as the baseline measurements at the start of the study. It has been shown that this is more robust than other approaches to dealing with missing data.

All this analysis will be carried out using the SAS statistical package.

### Legal and ethical considerations

This clinical trial will comply with the following regulations: the 2008 version of the Declaration of Helsinki, the Spanish Royal Decree 223/2004 of 6th February and the recommendations of the Council of Europe (Good Clinical Practices for Clinical Trials and Medicinal Products in the European Community, 17th January 1997). The protocol of the clinical trial has been agreed on by the primary care research committee, approved by the Clinical Research Ethics Committee of Euskadi, which is the reference committee, and authorised by the Spanish Agency for Medicaments and Health Products.

Only the researchers involved in the study will have access to patient codes. In relation to this, we will comply with the Spanish Act 14/2007 of Biomedical Research and Royal Decree 1720/2007 of 21 December that approves the regulations on the Development of the Organic Act 15/1999 of 13 December on Personal Data Protection.

### Limitations

One of the main potential limitations of this study is the difficulty of carrying out a triple-blind, placebo-controlled trial, with a drug that is already available in the market. This will be overcome by contracting the Laboratorium Sanitatis to prepare special formulations of the drug and the placebo.

The other possible limiting factor is the difficulty of effectively coordinating a multicentre trial in primary care. To address this issue, we will draw on the broad experience of the PCRUB in coordinating research projects across the network, namely within redIAPP and CAIBER.

## Discussion

The aim of this clinical trial is to assess the efficacy of a very easy-to-implement intervention for reducing blood pressure, namely just changing of the time of ASA administration in patients who are already taking this drug for secondary prevention. The findings could notably contribute to achieving good control of BP and, thereby, decrease cardiovascular risk among hypertensive patients; which remains an outstanding challenge. In the 2010 PRESCAP study, it was found that there was only good control of both systolic and diastolic BP in 46% of the study population even though, overall, >63% were on more than one type of antihypertensive drug [19,20]. There is a direct relationship between BP and the risk of suffering cardiovascular events, a 10% increase in BP increasing the risk of suffering cardiac events by 33% and of suffering from a CVA by 50% [8].

Several interesting studies have been published that show that night-time administration of low-dose ASA significantly reduces BP [1,3,4]. Notably, there is a more marked reduction in non-dippers, with night-time ASA even modifying the circadian pattern of BP by up to 58%, transforming them into dippers [2]. This last point is of great importance given that there is a direct relationship between the night-time dip in BP and the risk of suffering cardiovascular events, these being more common in non-dippers [11-14]. To date, all the studies have been conducted in healthy individuals or people with mild hypertension receiving no drug treatment [1-4]; although the results have been very promising, the effect of bedtime ASA on patients under antihypertensive treatment has not been demonstrated, hence the importance and relevance of undertaking this study.

Although there is no consensus over the use of ASA universally as primary prevention, [8,21,22] many hypertensive patients are under treatment with low-dose ASA as an antiplatelet agent, having previously had cardiovascular events [6-8]. Further, among patients with high CVR, there is a higher proportion of non-dippers than in the low-risk population [23]. The aforementioned studies suggest that BP could be reduced in patients already taking low-dose ASA as an antiplatelet therapy for secondary prevention, and that the reduction would be even greater with bedtime administration, that is, just by modifying the time of intake of ASA with no need to add extra drugs to their treatment, and that the magnitude of the reduction might transform non-dipper patients into dippers. Taking into account that it has been demonstrated that there is an association between a reduction in the night-time dip in BP and an increase in cardiovascular risk [11-14], we can expect that CVR will also decrease in these patients, with an improvement in the circadian pattern of BP.

In this study, we will use ABPM devices for a 24-h period, as the mean arterial pressure measured by this method is the measure that has been found to be most strongly correlated with the risk of morbidity and mortality and of suffering target organ damage due to hypertension [24-26]. In the design of the study, we have decided to take four sets of ABPM measurements. We understand that this could be considered inconvenient for patients, but we think this number of measurements is necessary to ensure the quality and reliability of the data. We hope that, given that the candidate patients have had a cardiovascular event, take several drugs (in most cases) and are concerned to optimise their control over risk factors, they will be willing to collaborate.

With respect to adverse effects, we do not expect to have more or different effects to those associated with the intake of low-dose ASA and this drug was already being taken by the participating patients prior to the trial. According to several studies [1-4], taking this drug in the evening does not increase the rate of adverse effects. Moreover, in a study that was carried out using 1,300 mg of ASA, it was found by endoscopy that there were 37% fewer cases of gastric bleeding when the ASA was taken at night than when it was taken in the morning [27].

We believe that our study will provide interesting data for the scientific community, as it is exploring new effects of a widely used substance. The greatest beneficiaries will, however, be patients who may potentially be able to achieve better control of the one of the most important cardiovascular risk factors and improve their prognosis, without having to take other drugs and with no further adverse effects. What is more, from the economic point of view, the treatment under trial does not represent an additional cost and could even lead to savings in healthcare; since when BP is reduced, there is a decrease in cardiovascular risk and, subsequently, in cardiovascular events and patients may require fewer antihypertensive drugs to achieve adequate control of their BP.

### Trial status

The status of the trial at submission is 104 patients recruited.

## Competing interests

The authors declared that they have no competing interests.

## Authors’ contributions

MVR, MCG and GG conceived the idea and are the study guarantors. They are primarily responsible for the study design and planning, obtained funding, and will be responsible for project coordination and supervision, analysis and interpretation of results and manuscript preparation. MCG and GG will be in charge of performing the analysis of results and critically reviewed the manuscript. NB will be the monitor of the study and EV and RSV will contribute to design the study and to obtain the target population. TAHPS group: RMP and HP participated in the supervision of team of Catalonia. VAA, MJOM, MLRI, MLJ, SAR, OEG, IAA, JFEA, IAB, MJBI, AAQ, EGR, JCMG, ATMR, LPE, VGU, IHM, MCGT, JBG, JAQV, GHI, JFZN, LMM, MBC, PTM, YCZ, JIM, XCC, MTAA, MDMV, ILC, MUBA, MCO, KAA, MAME, MCMGS, ATA, MLGA, ELG, ABI, JMJ, MAM, ARG, MSRA, AOM, ARM, MTGS, MMDFC, MCS, MV, MGA, JME, MM, UF, AN, ABB, NE, AND CM collaborated in the development of the protocol. All contributors have approved this version submitted for publication to *BMC Trials*. All authors read and approved the final manuscript.

## THAPS GROUP

Rosa Morros Pedros (RMP), Helena Pera (HP), Veronica Arce Arana (VAA), Mª Jose Oyarbide Mondaluce (MJOM), Mª Luz Rodríguez Ibáñez (MLRI), Mikel Luque Jiménez (MLJ), Santiago Ayechu Redín (SAR), Oihana Ezkurra Galdos (OEG), Iñigo Aguirre Arratibel (IAA), Jose Francisco Egido Arroyo (JFEA), Iosune Alberdi Buruaga (IAB), Mª Jesus Bernaras Iturrioz (MJBI), Amaia Arruti Querejeta (AAQ), Elisabete Gametxogoikoetxea Ribera (EGR), Juan Carlos Marlasca García (JCMG), Ana Teresa Martínez Rementería (ATMR), Loreto Palacio Elua (LPE), Violeta Gonzalez Urcelay (VGU), Itziar Hernando Mendibil (IHM), Mª Carmen Gonzalez Temprano (MCGT), Julián Bajo García (JBG), Jose Antonio Quindimil Vázquez (JAQV), Gabriel Hernández Iñarra (GHI), Jose Félix Zuazagoita Nubla (JFZN), Lucas Mengual Martínez (LMM), Mencía Benitez Camps (MBC), Pere Torán Montserrat (PTM), Yoseba Cánovas Zaldua (YCZ), Jordi Ingla Más (JIM), Xavier Cos Claramunt (XCC), Mª Teresa Amondarain Arratibel (MTAA), Mª Dolores Marin Vicuña (MDMV), Idoia Larrañaga -Cortabarria (ILC), Miren Uxue Busca Aguirre (MUBA), Magdalena Cuesta Ortiz (MCO), Kizkitza Alustiza Arteaga (KAA), Mª Antonia Machain Echarri (MAME), MªC Milagros Gómez Santamaría (MCMGS), Amaia Telleria Astarloa (ATA), Maria Luisa Garcia Aramaio (MLGA), Elena Lizaso Guerrico (ELG), Amparo Barrueco Iglesias (ABI), Josune Madariaga Jurrebaso (JMJ), Mercedes Armendáriz Múgica (MAM), Ana Roncero Gascón (ARG), Mª Soledad Romera Alegría (MSRA), Ana Ormaetxe Merodio (AOM), Azucena Ruiz Meléndez (ARM), Mª Teresa González Sanchoyerto (MTGS), María Mercedes Dolores Ferreras Castrillo (MMDFC), MªCecilia Salgueiro (MCS), Mónica Vera (MV), Marta Garcia Alfaro (MGA), Jose Manuel Escudero (JME), Montserrat Montoliu (MM), Ursula Flix (UF), Antonio Negrete (AN), Albert Boada Balmaceda (ABB), Nuria Esclans (NE) and Carlos Martin (CM).
